# Intersectionality as a tool for clinical ethics consultation in mental healthcare

**DOI:** 10.1186/s13010-024-00156-w

**Published:** 2024-05-02

**Authors:** Mirjam Faissner, Lisa Brünig, Anne-Sophie Gaillard, Anna-Theresa Jieman, Jakov Gather, Christin Hempeler

**Affiliations:** 1 Department of Psychiatry, Psychotherapy and Preventive Medicine, LWL University Hospital, Ruhr University Bochum, Bochum, Germany; 2 Institute of the History of Medicine and Ethics in Medicine, Charité - Universitätsmedizin Berlin, Thielallee 71, 14195 Berlin, Germany; 3 Institute for Ethics, History and Philosophy of Medicine, Hannover Medical School, Hannover, Germany; 4 Institute for Medical Ethics and History of Medicine, Ruhr University Bochum, Bochum, Germany; 5 Department of Biological and Experimental Psychology, School of Biological and Behavioural Sciences, Queen Mary University of London, London, UK

**Keywords:** Structural discrimination, Multiple discrimination, Social justice, Racism, Minority groups

## Abstract

Bioethics increasingly recognizes the impact of discriminatory practices based on social categories such as race, gender, sexual orientation or ability on clinical practice. Accordingly, major bioethics associations have stressed that identifying and countering structural discrimination in clinical ethics consultations is a professional obligation of clinical ethics consultants. Yet, it is still unclear how clinical ethics consultants can fulfill this obligation. More specifically, clinical ethics needs both theoretical tools to analyze and practical strategies to address structural discrimination within clinical ethics consultations. Intersectionality, a concept developed in Black feminist scholarship, is increasingly considered in bioethical theory. It stresses how social structures and practices determine social positions of privilege and disadvantage in multiple, mutually co-constitutive systems of oppression. This article aims to investigate how intersectionality can contribute to addressing structural discrimination in clinical ethics consultations with a particular focus on mental healthcare. To this end, we critically review existing approaches for clinical ethics consultants to address structural racism in clinical ethics consultations and extend them by intersectional considerations. We argue that intersectionality is a suitable tool to address structural discrimination within clinical ethics consultations and show that it can be practically implemented in two complementary ways: 1) as an analytic approach and 2) as a critical practice.

## Introduction

Major bioethics associations have emphasized the role of clinical ethics consultants in addressing structural discrimination within clinical ethics consultations (CEC). The American Society for Bioethics and Humanities, for instance, states in their *Code of Ethics and Professional Responsibilities for Healthcare Ethics Consultants*:

*When engaged in ethics consultation, consultants need to be attentive to the role that healthcare disparities, discrimination, and inequities play […] Consultants have a responsibility to identify and include relevant voices in the discourse, particularly marginalized voices. Recommendations of the consultation should not reinforce injustice* [1].

Bioethicists have recently particularly stressed the importance of considering race and racism^1^ in CEC and have developed first suggestions to address it [3–5]. When addressing racism, it is important to consider intersectionality. Intersectionality, a term coined by the legal scholar Kimberlé Crenshaw [6, 7], stresses the interdependency of multiple systems of discrimination, such as racism and sexism. Black feminist grassroots activists and scholars, particularly in the US, have highlighted the interconnectedness of oppressive social categories, for example, gender with race, age, sexual orientation and class, leading to unique social positions within a matrix of power dynamics [8]. Intersectionality stresses that not attending to this interconnectedness often leads to a marginalization of the experiences of multiply marginalized group members. Intersectionality is often understood both as an analytic lens or approach to better understand structural discrimination, and as a critical practice which aims at pursuing social justice [9].

Intersectionality is particularly useful as a framework for mental healthcare [10], because people experiencing mental illness are under a high risk of experiencing social stigmatization and discrimination based on mental illness, which may be compounded by further forms of discrimination [11]. Since discrimination is an important social determinant of mental health [12, 13], providing adequate mental healthcare services for people affected by structural discrimination is crucial. Yet, despite recent endeavors to eradicate discrimination towards minoritized individuals [14, 15], structural discrimination still impedes them from receiving adequate care and support [16]. As described in a systematic review on intersectionality and discrimination within mental healthcare, marginalized users may encounter stereotyping, microaggressions, and a lack of mental healthcare staffs’ knowledge and skills relevant to their care (e.g., regarding gender diversity, racism, or cultural humility) [11, 17]. Thus, while marginalized people are under a high mental health burden, mental healthcare is not sufficiently prepared for their needs, making intersectionality a particularly useful tool for mental healthcare. Clinical ethics consultants are important in this regard since they may both contribute to providing an anti-discriminatory care to multiply marginalized users as well as establish anti-discrimination within their own work.

In light of this, it seems crucial to consider intersectionality when attending to structural discrimination in CEC within mental healthcare. Yet, to our knowledge based on a systematic review on the use of intersectionality within bioethics, intersectionality has not yet been considered as a tool for CEC [18]. This article aims to fill this gap by examining the potentials of intersectionality – understood as both an analytic approach and a practical tool – to improve CEC. Because of the particularities of structural discrimination within mental healthcare highlighted above and our own experience within mental healthcare and psychiatric ethics, this article pays special attention to the context of mental healthcare. Nonetheless, we assume that our recommendations may also be applicable to other contexts of medical care. By analyzing the potential of intersectionality for CEC and by translating it into practical tools, we provide clinical ethics consultants with concrete suggestions to strengthen their anti-discrimination practice.

Our article is structured as follows: We start by outlining the typical structure and topics of CEC in mental healthcare to then give reasons why there is a need for anti-discrimination within CEC. Based on existing literature on anti-racist CEC and intersectionality, we propose two complementary ways in which intersectionality may inform and enrich CEC within mental healthcare: 1) as an analytic approach and 2) as a critical practice. We illustrate our suggestions based on a CEC case example adapted from a narrative ethics collection of clinical cases [19].

We acknowledge that our own social and academic backgrounds influence our work. It is thus important to mention that our research team is mainly White, with one Black woman, with backgrounds in psychiatry, psychology, gender studies, medical ethics, and philosophy. Our considerations were developed drawing on our experience with CEC within mental healthcare. 

## Current approaches to CEC in mental healthcare

CEC are a form of clinical ethics support provided by an individual or a small team to help identify, analyze and resolve ethical issues that arise in clinical practice [20, 21]. Although it is often open to requests from mental healthcare service users (henceforth: users) and relatives, it is requested mostly by clinicians or other healthcare personnel [22–24]. Typically, members of the mental healthcare team (e.g., nurses, psychiatrists, psychologists, social workers) and, if existent, the legal guardian of the user participate in a CEC. The participation of users in CEC is recommended [25] but still not the norm [26, 27]. For example, in one large German hospital, users participated in only 35% of CEC within mental healthcare between 2006 and 2015 [27].

Common ethical problems discussed in CEC include, for example, issues regarding patient autonomy, such as the applicability of advance directives or capacity to consent, treatment limitation and disagreements within the team regarding the appropriate course of treatment [22, 24, 27–29]. Within mental healthcare, some particular ethical problems are frequently discussed, for example, endangerment of self or others, declination of treatment suggestions, the use of coercive measures (e.g., physical or mechanical restraint) or involuntary treatment as well as questions around confidentiality [22, 24, 27, 29, 30]. The issues discussed in CEC in mental healthcare were seen as bearing a high risk of negatively affecting the therapeutic relationship [27].

Multiple methods for conducting CEC with no universally agreed standard exist [31, 32]. A commonly used method [33] that we also use in our own practice includes an opening where the reason for consultation is stated, information regarding the goals and procedure of the CEC is given, and all participants introduce themselves. This is followed by a collection of pertinent facts, which includes relevant medical, nursing, social and legal facts as well as the perspectives of users and relatives and other professionals involved. Afterwards, the decisional conflict is clarified, for example, “Is involuntary medication justified?” Drawing on normative frameworks, such as the four principles (i.e., respect for autonomy, beneficence, non-maleficence and justice) proposed by Beauchamp and Childress [34], the ethical conflict underlying the decision conflict is carved out. In the following discussion, ethical arguments are weighed up based on the concrete individual case. The aim is to jointly decide on an approach to solving the decisional conflict based on the chosen ethical framework, and to formulate concrete recommendations for the further proceeding. Ideally, the concrete recommendations are endorsed by all participants of the CEC. Finally, the results of the discussion are summarized and the clinical ethics consultant reviews whether a consensus could be reached.

Regarding the role of the clinical ethics consultant, three approaches are typically distinguished: The pure facilitation approach, the authoritarian approach, and the ethics facilitation approach [20, 35]. The pure facilitation approach proposes the clinical ethics consultant to act as a neutral mediator who focuses on facilitating a constructive dialogue and helps the participants find a consensus. Here, the clinical ethics consultant refrains from clarifying potentially useful concepts or ethical approaches, and takes no position on the relevant ethical values for the case. This differs from the authoritarian approach, in which the clinical ethics consultant pronounces what should be done and takes on moral decision-making authority. The approach we outlined above would be considered an ethics facilitation approach. In the ethics facilitation approach, the clinical ethics consultant clarifies the relevant normative concepts and offers assistance in the moral deliberation process while recognizing and conveying the boundaries for ethically acceptable solutions [20, 36].

## Why we need anti-discrimination within CEC in mental healthcare

There are two main reasons for anti-discrimination within CEC in mental healthcare: one normative and one epistemic. The normative reason is that clinical ethics consultants are under the moral obligation to support anti-discrimination by virtue of their professional role. To see why, note that according to the United Nations Committee on Economic, Social and Cultural Rights, the right to health is defined as “the right of everyone to the enjoyment of the highest attainable standard of physical and mental health” [37]. This implies that appropriate measures are taken to ensure healthcare services are accessible, both in law and in fact, without any discrimination on the grounds of “race, color, sex, language, religion, political or other opinion, national or social origin, property, birth, physical or mental disability, health status (including HIV/AIDS), sexual orientation and civil, political, social or other status” [37].

Yet, empirical research indicates that discriminatory practices continue to constitute an important barrier to the equal provision of mental healthcare [11]^2^ and occur both on the interpersonal and institutional level via organizational practices [16]. On the interpersonal level, overt racial prejudice may affect the therapeutic relationship [39, 40]. Further, research indicates that some user groups, e.g., women [41] or racially minoritized users [42], struggle to have their pain and symptoms acknowledged by doctors due to stereotypes. Moreover, Black users experience coercion, such as involuntary hospital admission, significantly more often than other users in mental healthcare [43]— a finding which Faissner and Braun [44] relate to the susceptibility to racist biases of the criteria used to ethically evaluate coercive interventions. CECs are part of clinical practice and, therefore, influenced by the same general dynamics as other clinical practices. Accordingly, different authors have suggested that discriminatory practices and biases may also play a role in the provision of CEC for marginalized users [3, 45]. For instance, CEC may be informed by patterns of prejudice and stereotyping based on explicit and implicit biases against racially minoritized service users, as suggested by Angove, Ngui and Repenshek [45].

Russell [46] suggests that both practitioners and clinical ethics consultants may start implementing anti-discrimination within healthcare institutions by considering the resources attached to their own professional role. Clinical ethics consultants play a crucial role in addressing structural discrimination within mental healthcare institutions. Since they provide guidance in concrete cases during CEC, create guidelines, and train staff as part of their institutional and professional responsibilities, clinical ethics consultants are in a good position to foster anti-discrimination – a commitment stressed by the Association of Bioethics Program Directors in their *Statement on Violence, COVID, and Structural Racism in American Society* [47].

The second reason for anti-discrimination within CEC is epistemic. As power structures are important in shaping clinical encounters and clinical decision-making, power imbalances and discriminatory practices in the hospital may contribute to ethical conflicts [48, 49]. Danis suggests that structural racism and biases may have a large explanatory role in ethical conflicts in the clinical context. For instance, some ostensibly “neutral” CEC on “disagreements between clinicians and patients, non-compliance [sic], difficulties with discharge planning, and resource allocation”, may be related to disadvantages and dynamics that are rooted in structural discrimination [5]. Additionally, some ethical conflicts that arise within the clinical setting may be grounded in structural processes that lie outside the clinical setting, for instance, in precarious living conditions or gendered violence. Considering structural factors, therefore, allows a better understanding of the core of an ethical conflict. These need to be considered in CEC in order to provide realistic recommendations [49].

Another facet of the epistemic reason for anti-discrimination within CEC are the epistemic costs through a loss of potentially relevant information due to marginalization and neglecting structural discrimination *within* the CEC. CEC may be understood as a knowledge practice in which the participants jointly develop a new understanding of an ethical problem. Philosophers analyzing questions around epistemic injustice have argued that power structures may lead to processes of *silencing* that affect the possibilities of marginalized users and staff to have their claims duly considered and participate in the interpretation of social experiences: *testimonial quieting* and *smothering* [50–52]. *Testimonial quieting *describes the unjustified downgrading of a person’s credibility based on stereotypes or implicit biases connected to the social identity of the speaker. For instance, Crichton, Carel and Kidd [51] argue that users’ contributions risk to be disbelieved or considered irrelevant in clinical practice, because, based on implicit biases, users may be considered incoherent, irrational or emotional by clinicians. Studies indicate that mental healthcare staff are not exempt from stereotypes against people experiencing mental illness.^3^ Additionally, both staff and users who are minoritized may face difficulties in being adequately heard in CEC. In clinical practice, a person’s social as well as professional position comes with different degrees of epistemic authority. For instance, a middle-aged White cis-male senior physician is very likely to be considered more authoritative in his views than a young Black female nurse – the picture gets more complex when considering various constellations within racial, gendered and professional hierarchies. *Testimonial* *smothering*, another form of silencing, arises when people decide to intentionally withhold information if they expect their communicative environment to lack the necessary skills to understand their claims. It has been suggested that clinical ethics committees that lack sensitivity regarding experiences of discrimination such as racism may discourage users and staff to share these experiences, which results in the loss of potentially important information [54].^4^ This is especially important for mental healthcare, where the invalidation of experiences of racism by White staff is reported as prevalent [11]. Given the normative and epistemic arguments for considering structural discrimination in CEC, we will next explain how intersectionality may be used as an analytic approach and a critical practice to address structural discrimination in CEC within mental healthcare.

## Applying intersectionality to clinical ethics

In order to provide concrete guidance on how to apply intersectionality within CEC, we introduce a clinical case example that we translated, adapted and simplified from a published narrative ethics collection of clinical cases based on our own practical experience [19]. We will draw on this case example to highlight how intersectionality can be used as an analytical approach and as a critical practice within CEC and exemplify different suggestions throughout the text.

### Case example

*The psychiatric staff of a locked psychiatric ward requests a CEC to discuss the further treatment of M. O. who has been admitted for the first treatment of a psychotic episode. She is introduced as a 23-year-old Muslim racially minoritized woman and described as rather short, neither slim nor obese, but very sporty.* 5

*M.O. refuses antipsychotic medication because she feels persecuted by the psychiatric team and fears being poisoned. During her stay, staff repeatedly considers M. O.’s behavior to be a threat to the safety of herself and others: she tries to escape the ward by climbing on stapled chairs at the back wall of the outdoor area, insults staff verbally and throws various objects at them. After repeated mechanical restraint, the psychiatrists suggest that if M. O. shows further instances of harm to others, restraint measures should be continued, and involuntary medication be considered. At this point, members of the team express the wish for clinical ethics support.*

### Intersectionality as an analytic approach

One core feature of intersectionality is to understand a given situation acknowledging the power structures that lead to positions of relative disadvantage and privilege [55]. It directs our attention to aspects that are obscured in analyses which are either oblivious to power structures or focus on a singular system of oppression and, thereby, disrupts our “deeply entrenched cognitive habits” [55]. We can, therefore, understand intersectionality as an *analytic approach* that is attentive to multiple social categories being equally salient in shaping practices and experiences within institutions. In the following, we will show how applying intersectionality as an analytic approach can lead to a more profound understanding of an ethical conflict within CEC.

Considering intersectionality as an analytic approach lends itself well to the clinical context because structural factors and discriminatory practices may play an important role in shaping ethical conflicts in clinical practice. Milliken and colleagues highlight that patients who decline treatment options are often perceived as “difficult” by the treatment team [48]. Yet, such negative characterizations of a patient may undermine the therapeutic process of finding realistic treatment options in line with the user’s preferences. Milliken and colleagues invite us to consider the underlying social and biographical aspects that may influence a person’s treatment preferences. An intersectional CEC should thus go beyond a mere discussion of an ethical conflict and take a step *back* to consider why an ethical conflict arose and how it is presented, considering structural factors.

When we think about the introduced case example, it is noteworthy that we do not learn much about the social and biographical factors of M. O., which are usually of high importance within mental healthcare. The way M. O. is presented to the clinical ethics consultants — the description of her body rather than her personal history and her own perspective on the situation — invites us to consider if structural processes of Othering or implicit biases may be involved in the conflict between M. O. and the team.^6^ Racially minoritized users, especially in the context of Anti-Muslim Racism in Germany, report Othering as one barrier to good care [57]. Research indicates that implicit biases about racialized users may influence the assessment of a person’s dangerousness, leading to the possible over-estimation of risk, especially in situations of high stress [44]. Such biases are enhanced when a person is not perceived as an individual with their personal history but rather as part of a social group. The description of M. O. suggests she might be constructed as a threat rather than a person in a mental health crisis needing care and attention.

During the CEC, the clinical ethics consultant should try to raise awareness about the role of structural racism in decisions about coercion. Experiencing racism in the healthcare system is known to reduce trust in the system [57]. Additionally, racially minoritized users may distrust psychiatric institutions due to its history of institutional racism and violence [58]. Yet, institutional trust appears important in deescalating the situation of repetitive coercion. The clinical ethics consultant may stipulate that racism may play a role in the conflict with M. O. and try to find out which, if any, anti-discriminatory actions have already been applied, e.g., measures of trust building, or creating a space to discuss experiences of discrimination that M. O. might have made within or outside the hospital. This may allow the clinical ethics consultant and the team to better understand the nature of the conflict and M. O.’s perspective on it. Considering these structural factors in the CEC may enhance the mutual understanding of the parties involved and open the perspective to further options. One way of initiating this process is the collection of intersectional facts (Table 1).

**Table 1:** Intersectional fact collection

Suggestion I – Intersectional fact collection
The collection of relevant clinical, social and legal facts is part of every CEC. Vo and Campelia [54] suggest noting how structural factors may play a role in each of these categories of facts to facilitate awareness of the pertinence of structural discrimination. Clinical ethics consultants may, as part of the standard procedure, ask questions such as 1. What role does the user’s social identity play? 2. Which power structures and systems of oppression are involved? 3. (How) may the conflict be related to structural discrimination? If possible, these questions may be considered together with the user, for example, with tools such as the *Structural Vulnerability Assessment Tool*, a tool recommended by MacDuffie and colleagues [59]. This assessment tool includes questions to assess the user’s financial security, residence, risk environments, food access, social network, legal status, education and previous experiences of discrimination. Another tool which allows to gather aspects of intersectional discrimination is the Intersectional Discrimination Index [60].

Using the information from the intersectional fact collection, clinical ethics consultants may encourage so-called “counter-storytelling,” a method emerging from narrative ethics and critical race theory [61]. Counter-storytelling refers to practices in which minoritized communities highlight their own experiences, stories or narratives that differ from the mainstream. In CEC, counter-storytelling allows the framing of an ethical conflict from the perspective of the user and/or relatives, while attending to social power structures. Intersectionality, due to its attentiveness to power relations within social and institutional practices, provides a helpful frame for building such counter-stories. To illustrate, in our example, a clinical ethics consultant could have suggested the counter-story of M. O.’s behavior as, for instance, a reaction to feelings of isolation and invalidation that are often described by users affected by discriminatory practices in mental healthcare [11]. Clinical ethics consultants may benefit from structural competency trainings to be able to engage in counter-storytelling (Table 2).

**Table 2:** Structural competency

Suggestion II – Structural competency
Clinical ethics consultants need structural competencies to facilitate a structural understanding within CEC via counter-storytelling. Metzl and Hansen [62] have developed a training for medical education called Structural Competency Training, which aims at promoting awareness about how individual level health outcomes, decisions and interactions result from structural level processes. It involves five core competencies, including 1) recognizing the structures that shape clinical interactions; 2) developing extra-medical conceptual tools to understand social structures; 3) replacing cultural framings of difference with structural frameworks; 4) observing and imagining structural interventions; and 5) developing structural humility, i.e., awareness about the limits of one’s own understanding and knowledge. The training has been adapted for mental healthcare [63] and clinical case descriptions [64]. In line with this, we strongly encourage an adaptation for clinical ethics training and advise clinical ethics consultants to engage with existing approaches in the meantime. Furthermore, completion of such training may be promoted by certifying structurally trained consultants.

Intersectionality not only provides a useful framework for understanding how the ethical conflict is shaped by social structures but also informs the ethical analysis itself. Discussing the decision conflict within a CEC is realized by choosing a fitting ethical framework and applying it to the ethical problem at hand. Different authors have argued that there is a conceptual White bias within medical ethics, including a strong focus on the four principles proposed by Beauchamp and Childress [34, 49, 65, 66]. It has been argued that these principles are not (yet) sensitive enough to account for discriminatory practices when analyzing ethical conflicts [67]. Furthermore, participants in CEC may hold ethical convictions and moral values that are not well captured by the four principles, for example, Confucian virtues, Indigenous values [49] or Islamic ethics [68]. Accordingly, braodening the ethical frameworks applied within CEC would be beneficial (Table 3). If a theoretical background is adopted by clinical ethics consultants without explicit reflection, this may contribute to marginalizing users’ – or other participants’ – normative values [54, 65]. Accordingly, clinical ethics consultants ought to make the choice of the ethical framework used in the CEC explicit and make sure it is acceptable to all the participants and individuals concerned. If clinical ethics consultants are unfamiliar with the users’ preferred ethical framework or values, epistemic humility, the awareness of their own limits of knowledge, is important. In our case example, the clinical ethics consultant should try to find out which values are important to M. O. and suggest a fitting ethical framework depending on M. O.’s preferences.

**Table 3:** Broadening ethical frameworks

Suggestion III – Broadening ethical frameworks
On a theoretical level, clinical ethics will benefit from broadening ethical frameworks. In this vein, a 2019 special issue in the American Journal of Bioethics on intersectionality [69] and a 2022 special issue in Bioethics on racism [70] are promising. Moreover, new approaches in clinical ethics, such as the transformative justice approach by Campelia and colleagues [49], illustrate new developments which are sensitive to underlying power structures. In terms of the four principles, it has been argued that intersectionality may be included in the principle of justice [67] or added as a fifth principle [71]. We encourage clinical ethics consultants to follow these debates.

Intersectionality encourages diversity in knowledge practices. It assumes that all people bring their own epistemic perspective, related to their social position, within complex social structures of privilege and oppression. This position shapes a person’s conditions of forming knowledge [9]. Especially if clinical ethics committees are very homogeneous regarding their members’ social positioning, e.g., in terms of race, gender, sexual orientation, age, ability, religion, cultural background or socioeconomic status, they may take some values for granted and be oblivious to how implicit value judgements shape the discussion. Problematically, implicit reliance on social values and background assumptions entails the risk of privileging some values at the expense of others. This risk may be reduced by diversifying clinical ethics committees (Table 4). Campelia and colleagues [49] invite clinical ethics consultants to critically reflect whether, and if so, why, certain values are privileged or suppressed. The reflection of one’s own position as well as questions of representation and diversity within clinical ethics committees are therefore highly important for intersectional CEC to address biases. In the presented case, it is important that the clinical ethics consultants are aware of their own stereotypical assumptions, e.g., based on gender, age, religion and race, given that M. O. is perceived as a young racially minoritized Muslim woman. If possible, the CEC should include the perspective of a racially minoritized woman and, only if the Muslim religion is relevant to M. O., someone with expertise in this field.

**Table 4:** Diversifying clinical ethics committees

Suggestion IV – Diversifying clinical ethics committees
On an organizational level, the diversity of the members of a clinical ethics committee may enhance the epistemic competency of the committee as different social positions come with different epistemic standpoints, conceptual know-how and experiential knowledge [5, 54, 72]. Magelssen and colleagues [73] suggest that ethics committees should include representatives from patient organizations or other types of counselling or self-help institutions, and argue that the inclusion of people who are not directly employed by the hospital may counter (implicit) biases towards the hospital’s interests. A careful composition of clinical ethics committees may thus allow for the identification and correction of biases.

### Intersectionality as a critical practice

Intersectionality can inform CEC and institutional practices not only by enhancing the understanding and analysis of an ethical problem but also by improving practices within CEC. It allows one to acknowledge the broader institutional and societal contexts in which clinical encounters occur [74] and act accordingly in pursuit of social justice [9]. As Hill Collins [9] stresses, intersectionality is *done* by various professions and we assume – as argued above – should be done by clinical ethics consultants. This encompasses three realms: 1) addressing power structures within the CEC, 2) addressing power structures within the hospital as an institution and 3) working towards social justice beyond the clinical setting. The focus of this article is on the first of these three aspects, but we will also mention the institutional structures necessary for anti-discrimination within CEC.

Understood as a critical practice, intersectional CEC aims to enhance the voice of users and make sure the space remains safe(r) for them and their representatives. To ensure that the user’s perspective is adequately represented and taken up within the CEC, clinical ethics consultants may have additional meetings with the user and representatives before and after the CEC. These may serve to warrant clinical ethics consultants understand the user’s perspective, and to identify and ideally address barriers to participation. Offering M. O. or someone supporting or representing her a meeting prior to the CEC may help to better understand her position, both in the hospital and in her everyday live.

*During* CEC, clinical ethics consultants are in a good position to make sure that all perspectives are equally heard, and to mediate between different perspectives and parties with special attention to power imbalances [3, 65, 75]. This may be achieved by introducing anti-discrimination moderation rules (Table 5). In order to open space to discuss the influence of racism in clinical practices, clinical ethics consultants should further use their moderation skills to unpack biases sometimes hidden in euphemisms and clinical technical terms, which should be omitted altogether to ensure that all participants can follow [76]. Moreover, clinical ethics consultants should make sure complaints about structural discrimination and racism are given appropriate uptake, and experiences are not questioned, trivialized or invalidated [59]. This involves identifying stereotypical assumptions, biases and microaggressions [77], i.e., short communicative interactions that convey a derogatory meaning and target members of marginalized social groups, during CEC.^7^ Beyond the epistemic costs of such practices (related to silencing and smothering as explained above), they fundamentally harm users in forms of emotional harms and in harms to one’s sense of self [77]. If instances of racism are dismissed in the CEC with M.O., the ethics consultant should emphasize the importance of recognizing that racism is prevalent in the clinical setting. They should highlight that individuals who are not directly impacted by racism may find it challenging to identify such experiences, and therefore, any complaints about racism should be treated seriously [59].

**Table 5:** Moderation rules

Suggestion V – Moderation rules
To enable all participants to participate equally in the CEC, clinical ethics consultants should pay special attention to meet the language needs of all participants, for example, ask everyone to speak loudly or slowly, use simple language, refrain from overreliance on medical terms or include a translator if necessary. Clinical ethics consultants should ensure that portions of speech are equal and use their moderation skills to interrupt if someone takes up a lot of space (or time) or ask others whether they want to share more of their perspective. In this regard, the American Society for Bioethics and Humanities endorses ethics consultants to „[u]se [their] own reflection to ensure that perspectives have been heard in proportion to the stake each voice has in the outcome of the discussion” [75]. Finally, clinical ethics consultants should clarify that discriminatory behavior will not be tolerated and will be reprimanded, if necessary, and that they will actively try to correct implicit biases and stereotypes.

Importantly, difficult situations can come up due to White fragility, i.e., negative feelings and reactions that arise out of “discomfort, defensiveness, or lack of familiarity with talking about race” [59]. Thus, clinical ethics consultants need sufficient literacy and awareness around racial issues to identify White fragility. Clinical ethics consultants need to be able to de-escalate such complex situations to protect the complainant. In cases of communicative breakdowns caused by power imbalances, lack of understanding or explicit discrimination, clinical ethics consultants need to enhance trust between the different parties by using their skills in “conflict resolution, mediation, negotiation” [3].

Problematically, clinical ethics consultants positioned in privileged social positions may lack skills to facilitate complex conversations with marginalized users [54]. They may, for instance, lack sufficient literacy for racial issues and epistemic sensitivity to recognize microaggressions [77]. Ethics consultants may thus benefit from further trainings, such as the Structural Competency Training (Table 2), or implicit bias trainings [79]. In the context of racism, Experiential Race Testimonies [80] competency trainings aiming at deconstructing Whiteness [81] are suggested.

Attending to power imbalances and practices of silencing or oppression within CEC may involve a critical reflection of the self-understanding and role of clinical ethics consultants. MacDuffie and colleagues argue that clinical ethics consultants should be neither neutral facilitators, nor take a soft advisory role, but should understand their role as *advocates* of users with marginalized social identities to balance power asymmetries [59]. In the context of discriminatory practices, a model in which the clinical ethics consultant takes responsibility for the outcome seems beneficial to prevent recommendations that further marginalize the user. This may be achieved within an ethics facilitation model, in which the clinical ethics consultant makes sure the recommendations are within the boundaries of anti-discrimination.

Beyond renegotiating the role of clinical ethics consultants, DiChristina [72] suggests understanding clinical ethics committees as Structural Justice Ethics committees that have the *explicit* function to advocate for social justice by ensuring the best clinical care for structurally marginalized communities within the hospital. Tasks may involve the gathering of data on discriminatory practices within the institution and preparing recommendations and guidelines for anti-discrimination organizational measures. This may also involve critically assessing whether the structures available to initiate a CEC and complain about discriminatory practices and health inequity concerns [59] are transparent to users. As most requests for CEC come from clinicians [22, 24, 28], informing users about the possibility of ethics support may enhance their agency in the clinical setting. Cooperation with the communities concerned, for example, through listening sessions (Table 6) and with existing bodies within the institution, such as Equal Opportunities Officers or staff offices that already collect such data, appear helpful for these organizational tasks.^8^

**Table 6:** Listening and learning sessions

Suggestion VI – Listening and learning sessions
In order to improve advocacy for marginalized communities, clinical ethics committees may get involved with members of marginalized social groups through “listening and learning sessions,” an idea also advanced by DiChristina [72]. In such sessions, clinical ethics consultants can learn about the concrete healthcare needs and barriers of specific communities, for example, trans* and gender nonconforming communities or communities of color. When organizing such meetings, the emotional burden and risk of re-traumatization when sharing difficult experiences need to be considered and mitigated through careful organization.

It is important to note that the institutional structures in which clinical ethics consultants’ work must be supportive for anti-discrimination within CEC to be possible. In a rapid evidence synthesis on research, policy recommendations, and examples from healthcare practice relevant to develop anti-racist healthcare practices, Jieman and colleagues stress the role of leadership to develop structures in which anti-racism is possible [82]. This is also relevant for clinical ethics consultants who rely on the support of the institutional leaders to be able to use their role-specific resources and possibilities to address discriminatory practices. To give just one example, clinical ethics consultants often can only contribute to institutional guidelines or clinical decisions if they are invited to do so. Thus, their work depends on the goodwill and acceptance of clinical and executive decision-makers. This creates a relationship of dependency which may limit their ability to challenge discriminatory practices [65]. These factors may, in turn, lead to clinical ethics consultants being reluctant to speak up against discriminatory practices if that would subvert existing power hierarchies [54], or they may fear retaliation by colleagues if they openly discuss racism [59]. In such constellations, expected “professional courtesy” may impede the criticism needed and clinical ethics consultants may reinforce, instead of challenge, existing power structures [65]. Thus, institutions and leadership need to claim their commitment to anti-discrimination and implement anti-discrimination measures to support conditions in which clinical ethics consultants can provide anti-discrimination within CEC. This may include employment strategies aiming at diversity and inclusion, staff training and education, reevaluating institutional practices and policies from an anti-discrimination perspective, putting in place structures to document and survey discrimination complaints, funding anti-discrimination, and establishing anti-discrimination as part of the institutional culture [82, 83].

## Concluding remarks

We have argued that structural discrimination has important effects on clinical practices that need to be taken seriously by clinical ethics consultants within their practice. As we have shown, intersectionality offers helpful insights into how CEC may support anti-discrimination. As an analytic approach, it offers a more nuanced understanding and analysis of ethical conflicts. As a critical practice, it allows one to address processes of marginalization within CEC. Given the dearth of empirical research on the applicability, acceptability and outcome of intersectional anti-discrimination within CEC, further research on conceptual questions as well as experiences with practice models is needed.

## Data Availability

Not applicable.

## Footnotes

1 Taking a social constructivist stance informed by critical race theory, we understand race as a social category which confers privilege and disadvantage based on physical features which are imagined to indicate a certain geographical ancestry [2]. Racism is a social system embedded in social structures, institutions and practices, in which privilege and disadvantage are distributed based on the category of ‘race’.

2 In a large national community-based survey on anti-Black racism in Germany, 65% of the participants who had used healthcare services in the last two years stated that they had been discriminated against. Most participants assumed that discrimination occurred based on skin color (74%), racism or “ethnic origin” (72%). The results of the survey showed that trans* and gender nonconforming individuals (with 83%) and cis-women (67%) were significantly more likely to report discrimination compared to cis-male participants (50%), as were individuals with disabilities (83%) in comparison to participants without disabilities (60%). This highlights the importance of attending to intersectional in-group differences [38].

3 For instance, according to a systematic review, mental healthcare staff hold the same level of stigma as the general population in the form of a wish for social distance towards people with a diagnosis of schizophrenia [53].

4 In the community-based survey on anti-Black racism in Germany referred to in footnote 2, 62% of the participants who responded to that item (*n* = 819) stated that psychotherapists did not take their experiences of racism seriously or invalidated them [38].

5 For argumentative reasons, we adopted this description from the original publication.

6 Othering describes social and discursive processes through which people are differentiated in in-group and out-group members. Out-group members are often constructed as inherently different and often inferior. The idea of Othering goes back to postcolonial theory, e.g., Said [56].

7 Microaggressions are termed “micro” because they occur on the interpersonal level or “microlevel.” They can be very subtle and are often overlooked by individuals who are not concerned. However, their effect is significant to the people affected [78].

8 MacDuffie and colleagues describe the successful cooperation of the ethics committee with the hospital’s Center for Diversity and Health Equity [59].

## References

[1] American Society for Bioethics and Humanities. Code of Ethics and Professional Responsibilities for Healthcare Ethics Consultants; 2014. Available from: https://asbh.org/uploads/ASBH_Code_of_Ethics.pdf. Cited 2023 Feb 24.

[2] Haslanger S, Haslanger S (2019). Tracing the sociopolitical reality of race. What Is Race?.

[3] Danis M, Wilson Y, White A (2016). Bioethicists can and should contribute to addressing racism. Am J Bioeth.

[4] Braddock CH (2021). Racism and bioethics: the myth of color blindness. Am J Bioeth.

[5] Danis M (2021). Putting anti-racism into practice as a healthcare ethics consultant. Am J Bioeth.

[6] Crenshaw K. Demarginalizing the Intersection of Race and Sex: A Black Feminist Critique of Antidiscrimination Doctrine, Feminist Theory and Antiracist Politics. University of Chicago Legal Forum 1989; 1989(1). Available from: URL: https://chicagounbound.uchicago.edu/uclf/vol1989/iss1/8.

[7] Crenshaw K (1991). Mapping the margins: intersectionality, identity politics, and violence against women of color. Stanford Law Review.

[8] Combahee River Collective. The Combahee River Collective Statement; 1977. Available from: URL: https://www.blackpast.org/african-american-history/combahee-river-collective-statement-1977/. Cited 2021 Oct 12.

[9] Hill CP (2015). Intersectionality's definitional dilemmas. Annu Rev Sociol.

[10] Funer F (2023). Admitting the heterogeneity of social inequalities: intersectionality as a (self-)critical framework and tool within mental health care. Philos Ethics Humanit Med.

[11] Hempeler C, Windel A-S, Schneider-Reuter L, Carlet J, Philipsen L, Juckel G, et al. Intersectionality and discriminatory practices in mental healthcare - a systematic review with qualitative evidence synthesis. Under submission. PROSPERO 2022. CRD42022284928. Available from: https://www.crd.york.ac.uk/prospero/display_record.php?ID=CRD42022284928. Cited 2024 March 22.

[12] Williams DR, Lawrence JA, Davis BA (2019). Racism and health: evidence and needed research. Annu Rev Public Health.

[13] Krieger N (2014). Discrimination and health inequities. Int J Health Serv.

[14] Mensah M, Ogbu-Nwobodo L, Shim RS (2021). Racism and mental health equity: history repeating itself. Psychiatr Serv.

[15] APA. APA's Apology to Black, Indigenous and People of Color for Its Support of Structural Racism in Psychiatry; 2018. Available from: URL: https://www.psychiatry.org/news-room/apa-apology-for-its-support-of-structural-racism. Cited 2022 Oct 25.

[16] Schouler-Ocak M, Bhugra D, Kastrup MC, Dom G, Heinz A, Küey L (2021). Racism and mental health and the role of mental health professionals. Eur Psychiatry.

[17] Mosher DK, Hook JN, Captari LE, Davis DE, DeBlaere C, Owen J (2017). Cultural humility: a therapeutic framework for engaging diverse clients. Pract Innov.

[18] Brünig L, Kahrass H, Salloch S. Intersectionality in Bioethical Debates. Review Protocol. 2021. Available from: osf.io/uw4xm. Cited 2024 Mar 22.

[19] Mandry C, Wanderer G, editors. Narrative Ethik in der Klinikseelsorge: Ethische und theologische Analysen und Diskussionen von Fallerzählungen. 1. Auflage. Stuttgart: Kohlhammer; 2023.

[20] Aulisio MP, Arnold RM, Youngner SJ (2000). Health care ethics consultation: nature, goals, and competencies. A position paper from the society for health and human values-society for bioethics consultation task force on standards for bioethics consultation. Ann Intern Med.

[21] Molewijk B, Slowther A, Aulisio M, ten Have H (2016). Clinical Ethics: Support. Encyclopedia of Global Bioethics.

[22] Wollenburg LM, Claus S, Kieser C, Pollmächer T (2020). [ The State of Application of Clinical Ethics Consultation in German Psychiatric Hospitals]. Psychiatr Prax.

[23] Orr RD, Shelton W (2009). A process and format for clinical ethics consultation. J Clin Ethics.

[24] Franke I, Speiser O, Dudeck M, Streb J (2020). Clinical ethics support services are not as well-established in forensic psychiatry as in general psychiatry. Front Psychiatry.

[25] Vorstand der Akademie für Ethik in der Medizin e. V (2023). Standards für Ethikberatung im Gesundheitswesen. Ethik in der Medizin.

[26] Fournier V, Rari E, Førde R, Neitzke G, Pegoraro R, Newson AJ (2009). Clinical ethics consultation in Europe: a comparative and ethical review of the role of patients. Clin Ethics.

[27] Löbbing T, Carvalho Fernando S, Driessen M, Schulz M, Behrens J, Kobert KKB (2019). Clinical ethics consultations in psychiatric compared to non-psychiatric medical settings: characteristics and outcomes. Heliyon.

[28] Ackermann S, Balsiger L, Salathé M (2016). Ethikstrukturen an Akutspitälern, Psychiatrischen Kliniken und Rehabilitationskliniken der Schweiz. Bioethica Forum.

[29] Syse I, Førde R, Pedersen R (2016). Clinical ethics committees – also for mental health care?. Norwegian Exp Clin Ethics.

[30] Montaguti E, Schürmann J, Wetterauer C, Picozzi M, Reiter-Theil S (2019). Reflecting on the reasons pros and cons coercive measures for patients in psychiatric and somatic care: the role of clinical ethics consultation. Front Psychiatry.

[31] Rasoal D, Skovdahl K, Gifford M, Kihlgren A (2017). Clinical ethics support for healthcare personnel: an integrative literature review. HEC Forum.

[32] Neitzke G. Formen und Strukturen Klinischer Ethikberatung. In: Vollmann J, Schildmann J, Simon A, editors. Klinische Ethik: Aktuelle Entwicklungen in Theorie und Praxis. Frankfurt New York: Campus; 2009. p. 37–56.

[33] Vollmann J, Dörries A, Neitzke G, Simon A, Vollmann J (2008). Methoden der ethischen Falldiskussion. Klinische Ethikberatung: Ein Praxisbuch.

[34] Beauchamp TL, Childress JF (2019). Principles of biomedical ethics.

[35] Tarzian AJ (2013). Health care ethics consultation: an update on core competencies and emerging standards from the American Society For Bioethics and Humanities' core competencies update task force. Am J Bioeth.

[36] Gasparetto A, Jox RJ, Picozzi M (2018). The notion of neutrality in clinical ethics consultation. Philos Ethics Humanit Med.

[37] Committee on economic, social and cultural rights. CESCR General Comment No. 14: The Right to the Highest Attainable Standard of Health (Art. 12); 2000. Available from: URL: http://docstore.ohchr.org/SelfServices/FilesHandler.ashx?enc=4slQ6QSmlBEDzFEovLCuW1AVC1NkPsgUedPlF1vfPMJ2c7ey6PAz2qaojTzDJmC0y%2B9t%2BsAtGDNzdEqA6SuP2r0w%2F6sVBGTpvTSCbiOr4XVFTqhQY65auTFbQRPWNDxL. Cited 2023 Apr 25.

[38] Aikins MA, Bremberger T, Aikins J. Afrozensus 2020: Perspektiven, Anti-Schwarze Rassismuserfahrungen und Engagement Schwarzer, afrikanischer und afrodiasporischer Menschen in Deutschland, Berlin; 2021. Available from: www.afrozensus.de. Cited 2023 Feb 24.

[39] Moore C, Coates E, Watson A’R, de Heer R, McLeod A, Prudhomme A (2023). "It's important to work with people that look like me": black patients' preferences for patient-provider race concordance. J Racial Ethnic Health Disparities.

[40] Goode-Cross DT, Grim KA (2016). An Unspoken Level of Comfort. J Black Psychol.

[41] Hoffman KM, Trawalter S, Axt JR, Oliver MN (2016). Racial bias in pain assessment and treatment recommendations, and false beliefs about biological differences between blacks and whites. Proc Natl Acad Sci U S A.

[42] White AA (2014). Some advice for minorities and women on the receiving end of health-care disparities. J Racial Ethnic Health Disparities.

[43] Barnett P, Mackay E, Matthews H, Gate R, Greenwood H, Ariyo K (2019). Ethnic variations in compulsory detention under the mental health act: a systematic review and meta-analysis of international data. Lancet Psychiatry.

[44] Faissner M, Braun E. The ethics of coercion in mental healthcare: the role of structural racism. J Med Ethics. 2023:108984. Online ahead of print. 10.1136/jme-2023-108984 PMC11228209 37845011

[45] Angove RSM, Ngui EM, Repenshek M. Inclusion and use of race and Ethnicity in ethics consultation research; 2014. Available from: https://www.chausa.org/publications/health-care-ethics-usa/archives/issues/fall-2014/inclusion-and-use-of-race-and-ethnicity-in-ethics-consultation-research. Cited 2023 Feb 24.

[46] Russell C (2022). Meeting the moment: bioethics in the time of black lives matter. Am J Bioeth.

[47] Association of Bioethics Program Directors. ABPD Statement on violence, COVID, and structural racism in American Society; 2020. Available from: URL: https://www.bioethicsdirectors.net/abpd-statement-on-violence-covid-and-structural-racism-in-american-society/ . Cited 2023 Feb 24.

[48] Milliken A, Jurchak M, Sadovnikoff N (2018). When societal structural issues become patient problems: the role of clinical ethics consultation. Hastings Cent Rep.

[49] Campelia G, Olszewski AE, Brazg T, Vo HH (2022). Transformative justice in ethics consultation. Perspect Biol Med.

[50] Carel H, Kidd IJ (2014). Epistemic injustice in healthcare: a philosophial analysis. Med Health Care Philos.

[51] Crichton P, Carel H, Kidd IJ (2017). Epistemic injustice in psychiatry. BJPsych Bull.

[52] Dotson K (2011). Tracking epistemic violence, tracking practices of silencing. Hypatia.

[53] Valery K-M, Prouteau A (2020). Schizophrenia stigma in mental health professionals and associated factors: a systematic review. Psychiatry Res.

[54] Vo H, Campelia GD (2021). Antiracist activism in clinical ethics: what's stopping us?. Hastings Cent Rep.

[55] Carastathis A (2014). The concept of intersectionality in feminist theory. Philos Compass.

[56] Said EW (2021). Orientalism.

[57] DeZIM. [Racism and its symptoms. Report of the National Monitoring of Discrimination and Racism with a focus on health]; 2023. Available from: https://www.dezim-institut.de/fileadmin/user_upload/Demo_FIS/publikation_pdf/FA-5824.pdf. Cited 2023 Dec 19.

[58] Metzl JM (2011). The protest psychosis: How schizophrenia became a black disease.

[59] MacDuffie KE, Patneaude A, Bell S, Adiele A, Makhija N, Wilfond B (2022). Addressing racism in the healthcare encounter: The role of clinical ethics consultants. Bioethics.

[60] Scheim AI, Bauer GR (2019). The Intersectional Discrimination Index: Development and validation of measures of self-reported enacted and anticipated discrimination for intercategorical analysis. Soc Sci Med.

[61] Olszewski AE, Scott M, Patneaude A, Weiss EM, Wightman A (2021). Race and power at the bedside: counter storytelling in clinical ethics consultation. Am J Bioeth.

[62] Metzl JM, Hansen H (2014). Structural competency: theorizing a new medical engagement with stigma and inequality. Soc Sci Med.

[63] Metzl JM, Hansen H (2018). Structural competency and psychiatry. JAMA Psychiat.

[64] Hassan IF, Bui T (2022). The structural analysis: incorporating structurally competent clinical reasoning into case-based presentations. J Gen Intern Med.

[65] Ho A (2016). Racism and bioethics: are we part of the problem?. Am J Bioeth.

[66] Myser C (2003). Differences from somewhere: the normativity of whiteness in bioethics in the United States. Am J Bioeth.

[67] Rogers J, Kelly UA (2011). Feminist intersectionality: bringing social justice to health disparities research. Nurs Ethics.

[68] Mustafa Y (2014). Islam and the four principles of medical ethics. J Med Ethics.

[69] Cheema AW, Meagher KM, Sharp RR (2019). Multiple marginalizations: what bioethics can learn from black feminism. Am J Bioeth.

[70] Ganguli-Mitra A, Shahvisi A, Ballantyne A, Ray K (2022). Racism in healthcare and bioethics. Bioethics.

[71] Grzanka PR, Brian JD, Shim JK (2016). My bioethics will be intersectional or it will be bleep. Am J Bioeth.

[72] DiChristina W. Structural justice ethics in health care. VIB. 2021;7.10.52214/vib.v7i.8404.

[73] Magelssen M, Pedersen R, Førde R (2014). Sources of bias in clinical ethics case deliberation. J Med Ethics.

[74] Barned C, Lajoie C, Racine E (2019). Addressing the practical implications of intersectionality in clinical medicine: ethical, embodied and institutional dimensions. Am J Bioeth.

[75] American Society for Bioethics and Humanities. Resources for developing advanced skills in ethics consultation; 2017. Available from: https://asbh.org/uploads/publications/Resources_for_Ethics_Consultation.pdf. Cited 2024 Jan 19.

[76] Dudzinski DM (2018). White Privilege and Playing It Safe. Am J Bioeth.

[77] Freeman L, Stewart H (2018). Microaggressions in clinical medicine. Kennedy Inst Ethics J.

[78] Sue DW, Capodilupo CM, Torino GC, Bucceri JM, Holder AMB, Nadal KL (2007). Racial microaggressions in everyday life: implications for clinical practice. Am Psychol.

[79] Fuller LL (2016). Policy, advocacy, and activism: on bioethicists' role in combating racism. Am J Bioeth.

[80] Ray KS (2021). Going beyond the data: using testimonies to humanize pedagogy on black health. J Med Humanit.

[81] Lazaridou F, Fernando S (2022). Deconstructing institutional racism and the social construction of whiteness: a strategy for professional competence training in culture and migration mental health. Transcult Psychiatry.

[82] Jieman A-T, Onwumere J, Woodhead C, Stanley N, Hatch SL. Developing anti-racist practice to support Black and other racial minority nurses and midwives within the NHS: a rapid qualitative evidence synthesis; 2022. Available from: URL: https://tidesstudy.com/developing-anti-racist-practice-to-support-black-and-other-racial-minority-nurses-and-midwifes-within-the-nhs-a-rapid-qualitative-evidence-synthesis-full-report/. Cited 2024 Jan 19.

[83] South EC, Butler PD, Merchant RM (2020). Toward an equitable society: building a culture of antiracism in health care. J Clin Invest.

